# Comparison of primary liver cancer mortality estimates from World Health Organization, global burden disease and global cancer observatory

**DOI:** 10.1111/liv.15357

**Published:** 2022-07-09

**Authors:** Chenxi Li, Wen‐Qiang He

**Affiliations:** ^1^ Melbourne School of Population & Global Health The University of Melbourne Melbourne Australia; ^2^ School of Population Health UNSW Sydney Sydney Australia; ^3^ Childrens Hospital at Westmead Clinical School The University of Sydney Sydney Australia

**Keywords:** age‐standardized rate, average annual percentage change, completeness of cause‐of‐death registry, global burden disease, global cancer observatory, mortality, World Health Organization

## Abstract

**Aims:**

This study aims to compare estimates of primary liver cancer mortality from World Health Organization (WHO), Global Burden Disease (GBD) and Global Cancer Observatory (GCO).

**Methods:**

Liver cancer mortality was extracted from WHO, GBD and GCO for 92 countries for the most recent year. Age‐standardized rate (ASR) was computed and used for current comparisons across the three data sources. Temporal trend for 75 countries was analysed and compared between WHO and GBD from 1990 to 2019 using joinpoint regression. Average annual percentage change for the most recent 10 years was used as indicator for change.

**Results:**

The estimates of ASR were quite consistent across the three data sources, but most similar estimates were found between WHO and GCO in both region and country levels. The differences in ASR were negatively correlated with completeness of cause‐of‐death registration, human development index and proportion of liver cancer because of alcohol consumption. Consistent trends of ASR were found from 35 countries between WHO and GBD in the most recent 10 years. However, opposite trends were found from 10 countries with five from Southern America, four from Europe and one from Asia. Of the 18 countries for projection, opposite trends between WHO and GBD were found from seven countries.

**Conclusion:**

While the ASR of primary liver cancer mortality was comparable across the three data sources, most similar estimates were found between WHO and GCO. The opposite trends found from 10 countries between WHO and GBD raised concerns of true patterns in these countries.

## Lay summary

To date, the global estimates of primary liver cancer mortality have been provided by World Health Organization, Global Burden Disease, and Global Cancer Observatory, yet no study has compared the current estimates of primary liver cancer mortality from different data sources globally. This study aims to compare the most recent estimates of primary liver cancer mortality from 92 countries across these three sources. The comparisons were also made through the temporal trend analysis from 1990 to 2019, and the projection to 2030 between WHO and GBD. While the estimates of primary liver cancer mortality were comparable across the three data sources for 92 countries, most similar estimates were found between WHO and GCO. Opposite trends in the most recent 10 years were identified from five Southern American countries (Colombia, Cuba, Dominican Republic, Jamaica and Mexico), four European countries (Latvia, Sweden, Cyprus and Poland) and one Asian country (Uzbekistan).

**Abbreviations**
AAPC: average annual percentage change
APC: average percentage change
ASR: age‐standardized rate
GBD: global burden disease
WHO: world health organization

## 1 BACKGROUND

Primary liver cancer is the sixth most commonly diagnosed cancer and the third leading cause of cancer death worldwide in 2020, accounting for approximately 906 000 new cases and 830 000 deaths.^1^ With the universal vaccination against hepatitis B virus (HBV) and administration of highly effective antiviral treatment for HBV and hepatitis C virus (HCV), significant decrease in liver cancer has been reported in some high‐risk countries in Eastern and South‐Eastern Asia.^2^, ^3^ However, incident rates in formerly low‐risk countries including Japan, Australia, Europe, Canada and the United States have been increasing in the last 20 years.^1^

To date, the global estimates of primary liver cancer mortality have been provided by three major international groups, the Mortality Database by the World Health Organization (WHO), the Global Burden Disease (GBD) by the Institute for Health Metrics and Evaluation (IHME), and the Global Cancer Observatory (GCO) by the International Agency for Research on Cancer (IARC). While the WHO Mortality Database was based on submitted data from each national/regional death registry,^4^ the mortality data from both GBD and GCO were estimated using some statistical modelling based on various data sources.^1^, ^5^ Estimates for health data reflect massive initiatives by some of the world's experts, yet the results for country‐level estimates are dramatically different between different groups.^6^, ^7^ Previous analyses have also shown that comparative studies of global health estimates are critical to prioritize health policy and to highlight less reliable estimates and data gaps in specific countries.^6^, ^8^ To our knowledge, no study has compared the current estimates of primary liver cancer mortality across different data sources globally.

Previous studies have investigated the temporal trends of primary liver cancer mortality using estimates from WHO and GBD.^9^, ^10^ Although the overall trends between them were similar for some countries with high risk of viral hepatitis in Eastern Asia (Japan and South Korea), variations were found across countries from other continents.^9^, ^10^ Some of the trends were contradictory with large differences between these two sources. For instance, significant reduction was found from Norway, Finland and France from the study based on the WHO Mortality Database from 2000 to 2012^9^ while increase was found from the other study based on GBD Study data in the same period.^10^ In addition, the study based on WHO Mortality Database only selected 28 countries to report the mortality rate with the majority of them from Europe and the study only selected 11 countries to show their trends over time.^9^ However, no study has compared the temporal trends of primary liver cancer mortality between them globally.

This study, therefore, aims to assess and compare the national and regional burden of liver cancer from 92 selected countries using most recent estimates of primary liver cancer deaths from WHO, GBD and GCO. We also made comparison of temporal trends from 1990 to 2019 for 75 countries and comparison of mortality projection up to 2030 for 18 countries between WHO and GBD.

## 2 METHODS

### 2.1 Data sources

Mortality of primary liver cancer was extracted from the WHO Mortality Database.^4^ The WHO Mortality Database comprises deaths registered in national/regional vital registration systems, with underlying cause of death as coded by the relevant national authority. Only medically certified deaths were published by WHO Mortality Database. The database contains number of deaths by country, year, sex, age group, cause of death and the total number of populations by age and sex. Data are included only for countries reporting data properly coded according to the International Classification of Diseases (ICD). A total of 92 countries from 9 regions were selected as they reported at least 10 cases from or after 2014 and 75 of them were included for trend analysis as they provided consistent data with at least 10 cases each year from 1990 to 2019. During the study period, two different revisions of the International Classification of Diseases (ICD) were used, including ICD‐9 code 155.0–155.9 and ICD‐10 C22.0‐C22.9 for primary liver cancer from WHO Mortality Dataset. However, due to substantial variations of estimates from the transition year of ICD‐9 to ICD‐10 for some countries, we only considered the years since the adoption of ICD‐10 for the following countries: France, Japan, South Korea, Dominican, El Salvador, Guatemala, Jamaica, Paraguay, Puerto Rico and Chile.

Primary liver cancer mortality data from the GBD 2019 Study was retrieved for the same 92 countries for the same year and 75 of them for the same calendar years through Global Health Data Exchange query tool.^11^ ICD‐9 code 155.0–155.9 and ICD‐10 codes C22.0–22.9 were used for primary liver cancer over the period. The estimation process for primary liver cancer deaths has been described from the previous study.^5^ Data sources for the estimation include WHO vital registration (83% of data), cancer registry (14% of data), and verbal autopsy data and literature review (3% of data).^12^ If mortality data were not available, incidence data were transformed to mortality estimates using separately modelled incidence‐to‐mortality ratios. Data were adjusted for age groups, aggregated causes, uninformative causes of death and modelled by developing a large set of plausible models using different model types and combinations of covariates.^13^ Aggregated causes of liver cancer included alcohol consumption, HBV, HCV, mean body mass index, tobacco, diabetes and uninformative causes included education, health system access and socioeconomic index.^13^ GBD also generated its own population estimates for each country.^5^

Estimates of primary liver cancer mortality for the most recent year (i.e. 2020) was also obtained from GCO for the same 92 countries.^14^ ICD‐10 code C22.0‐C22.9 was used for primary liver cancer for year 2020. Estimates were either based on projections from WHO Mortality Database, extrapolates from neighbouring countries, or modes from incidence‐to‐mortality ratios as described previously.^1^ National population estimates for 2020 were extracted from the United Nations website.^15^

To explore potential reasons for the difference in mortality estimates across WHO, GBD and GCO, the following variables were investigated: the completeness of cause‐of‐death registration from WHO,^16^ human development index (HDI) from United Nations,^17^ proportions of primary liver cancer attributable to HBV, HCV, alcohol consumption and NASH from GBD,^11^ and data sources used for estimation by GCO.^14^ We also collected Socio‐Demographic Index (SDI) data at the national level from GBD.^18^

### 2.2 Analysis

The current burden of primary liver cancer mortality was investigated and compared using number of deaths and age‐standardized rates (ASR) of death in the most recent year. Depending on the availability of mortality data, most of the countries provided number of deaths in 2019 as the most recent year from WHO (*n* = 57), while some other countries had data from 2014 to 2018 as the most recent year (*n* = 38). Estimates in number of deaths for the most recent year were selected for the same year from WHO and GBD, but all the estimates from GCO were from year 2020. The number of deaths for each region was computed by summing the total number of deaths in each country within the region. ASR has been calculated for each country across the three data sources based on the same standard population. For this, direct method has been used based on the 2000 world population in 5‐year age groups as the standard for each country and region as described previously.^3^ The pairwise difference in number of deaths and ASR in the most recent year for each country and region was computed as the pairwise absolute difference between two estimates divided by the mean of the two estimates from any two of the three data sources. To investigate the pairwise difference, countries were also grouped by their SDI into Low‐SDI (0–0.45), Middle‐Low SDI (0.45–0.61), Middle SDI (0.61–0.69), High‐Middle SDI (0.69–0.81) and High SDI (0.81–1.00).^18^

In addition, to assess the impact of including different years of estimates across the three data sources, a sensitivity analysis was conducted for the comparison of ASR from 21 selected countries where WHO Mortality data were available for the year 2020 with estimates from GBD in the year 2019 and GCO in the year 2020. Similarly, the estimates of ASR were calculated and pairwise difference was applied for comparisons.

Pearson correlation was used to investigate the correlation of ASR from each data source and the correlation of the differences in ASR across the three data sources with potentially explanatory variables mentioned above. Pearson correlation coefficients (*r*) were calculated to show the strength of the relationship and linear regression lines were plotted for each association. ANOVA was used for the comparisons of ASR and pairwise difference of ASR through the three data sources.

As the GCO used the same estimates of primary liver cancer deaths as those from WHO for the period of 1990 to 2019, the temporal trend analysis was only conducted based on estimates from WHO and GBD using joinpoint regression. Joinpoint regression analysis was used to identify years when a significant change of ASR in the linear slope of the temporal trend occurred over the period with a maximum of two joinpoints. The estimated annual percent change was then computed for each of the identified trends by fitting a regression line to the natural logarithm of the rates using calendar year as a regressor variable. We calculated the average annual percent change (AAPC) over the last 10 years, based on an underlying joinpoint model, to show the most recent trend. The trend was considered as significant reduction if AAPC and its upper estimate of 95% were less than 0 and it was considered as significant increase if AAPC and its lower estimate of 95% were greater than 0. Otherwise, the trend was considered as no change or stable over the period.^19^ The results from the joinpoint analysis were then compared between WHO and GBD based on AAPC. Similar trend was defined as the same direction of change from the two comparisons. Opposite trend was defined as opposite direction of change from the two comparisons.

For the top 18 countries with the highest death number of primary liver cancer in the most recent year, estimates of mortality were projected to 2030. For this, we applied a logarithmic Poisson joinpoint regression model to each 5‐year age‐specific number of deaths, setting a maximum of two joinpoints, to identify the most recent trend segment. The detail of the projection was described as previously.^9^ In brief, we estimated age‐specific numbers of deaths and the corresponding 95% prediction intervals (PIs) for 2030 by fitting a linear regression to the mortality data from each age‐group over the most recent trend segment identified by the joinpoint model. We computed predicted age‐specific and age‐standardized death rates with 95% PIs using predicted age‐specific numbers of deaths obtained from our model and predicted populations from United Nations.^15^

## 3 RESULTS

Table 1 shows the key differences in estimates of primary liver cancer mortality by data sources and modelling methods from WHO, GBD and GCO. The WHO mortality data were based on submitted data by each member country to WHO by 5‐year age groups and sex. No modelling methods have been used for WHO mortality data. In contrast, the estimates from the GBD study were based on various sources of data including WHO mortality data, cancer registry and verbal autopsy. After adjusting these data with some covariates using Cause of Death Ensemble model (CODEm), the GBD estimates were obtained by feeding these adjusted data to a Bayesian meta‐regression modelling tool, DisMod‐MR 2.1. However, the details of input data for each country were not publicly available although the output data were provided overall, by 5‐year age groups, sex and aetiology for 204 countries. As for estimates from GCO, they were also based on few different methods depending on the availability and quality of registration data, but no adjustments were made for the estimation. The details of input data were published for each country and estimates were available overall, by age and sex for 185 countries.

**TABLE 1:** Characteristics of data and methodology for country‐specific primary liver cancer mortality estimates from WHO, GBD and GCO

Overall model strategy	WHO mortality dataset	GBD estimates	GCO estimates
Data			
Data sources included for the dataset	WHO vital registration	WHO vital registration, cancer registry data, verbal autopsies and peer‐reviewed literature	WHO vital registration, cancer registry data and neighbouring countries
Data stratified by age	Yes	Yes	Yes
Number of age groups	20	18	15
Data stratified by sex	Yes	Yes	Yes
Population	Each Member	GBD estimates	WHO
Included countries	140	204	185
Period	1942–2020	1990–2019	2020
Quality assessment	Assessed representativeness of study data	No	No
Modelling methods			
Statistical/Modelling methods applied	No	Cause of death ensemble approach, mixed‐effects regression	Countries with mortality data: short‐term prediction models; for countries without national data: Incidence‐to‐mortality ratios
Extrapolation for missing data	No	Yes	Selected neighbouring countries
Model covariates	No	Yes	No
Uncertainty incorporated	No	Yes	Yes

Abbreviations: GBD, Global Burden Disease; GCO, Global Cancer Observatory; WHO, World Health Organization.

**FIGURE 1:**
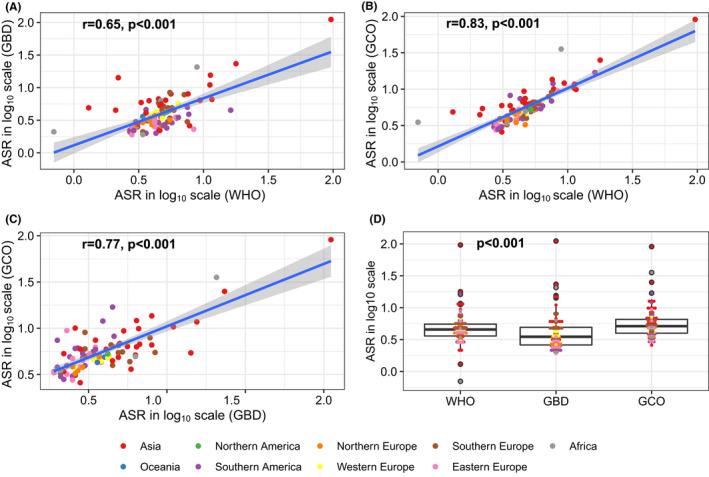
Correlations and comparisons of age‐standardized rates of primary liver cancer mortality from 92 selected countries between the pairs among WHO, GBD and GCO. WHO, World Health Organization; GBD, Global Burden Disease; GCO, Global Cancer Observatory; ASR, age‐standardized rate.

Of the 92 countries included for current burden comparison, higher correlation of ASR was found between WHO and GCO (*r* = 0.83, *p* < 0.001) than that between WHO and GBD (*r* = 0.65) and between GBD and GCO (*r* = 0.77) (Figure 1). Overall, ASR from GBD were slightly lower than those from WHO and estimates from GCO were slightly higher than those from WHO but without statistical significance. In country‐specific estimate, the top three countries with the highest ASR of primary liver cancer deaths were Mongolia, Thailand and Egypt (Table 2).

**TABLE 2:** Number of death and age‐standardized rates (ASR) of primary liver cancer mortality for 92 selected countries in 2019 (or as indicated in the bracket) and the pairwise differences in ASR from WHO, GBD and GCO

Region/country	WHO		GBD		GCO		Pairwise difference in no. of death (%)			Pairwise difference in ASR (%)		
	No. of death	ASR	No. of death	ASR	No. of death	ASR	WHO/GBD	WHO/GCO	GBD/GCO	WHO/GBD	WHO/GCO	GBD/GCO
**Asia**	71 890	7.8	93 537	9.8	98 118	10.3	26.2	30.9	4.8	22.7	27.6	5.0
Armenia	462	11.6	290	6.6	414	9.9	45.7	11.0	35.2	54.9	15.8	40.0
Brunei Darussalam	32	11.2	35	11.0	35	10.3	9.0	9.0	0.0	1.8	8.4	6.6
Iran (Islamic Republic of) (2016)	3142	4.7	2348	3.5	5324	7.4	28.9	51.5	77.6	29.3	44.6	71.6
Iraq (2016)	557	3.3	1213	5.9	686	3.6	74.1	20.8	55.5	56.5	8.7	48.4
Israel (2018)	333	2.8	315	2.6	360	3.1	5.6	7.8	13.3	7.4	10.2	17.5
Japan	25 264	5.6	34 514	8.0	28 155	6.6	30.9	10.8	20.3	35.3	16.4	19.2
Jordan (2015)	117	2.7	103	2.2	197	3.4	12.7	51.0	62.7	20.4	23.0	42.9
Kazakhstan	702	3.8	1116	6.3	988	5.1	45.5	33.8	12.2	49.5	29.2	21.1
Kuwait	56	4.6	54	2.2	122	5.3	3.6	74.2	77.3	70.6	14.1	82.7
Kyrgyzstan	347	7.8	118	2.6	465	10.0	98.5	29.1	119.0	100.0	24.7	117.5
Lebanon (2017)	147	3.1	148	2.8	168	2.6	0.7	13.3	12.7	10.2	17.5	7.4
Libya (2017)	43	1.3	234	4.9	213	4.9	137.9	132.8	9.4	116.1	116.1	0.0
Malaysia (2014)	909	4.5	1540	6.7	2050	6.7	51.5	77.1	28.4	39.3	39.3	0.0
Maldives	11	4.6	17	5.3	28	9.4	42.9	87.2	48.9	14.1	68.6	55.8
Mongolia (2016)	1741	96.2	2140	111.1	2060	90.7	20.6	16.8	3.8	14.4	5.9	20.2
Oman	35	2.1	83	4.5	121	4.4	81.4	110.3	37.3	72.7	70.8	2.2
Philippines	6401	9.4	5276	6.4	9953	12.1	19.3	43.4	61.4	38.0	25.1	61.6
Qatar	22	2.2	91	14.2	54	5.4	122.1	84.2	51.0	146.3	84.2	89.8
Singapore	531	7.6	658	8.0	1270	13.6	21.4	82.1	63.5	5.1	56.6	51.9
South Korea	10 586	11.3	14 477	15.5	11 158	11.7	31.0	5.3	25.9	31.3	3.5	27.9
Tajikistan (2017)	160	3.6	177	4.1	291	5.9	10.1	58.1	48.7	13.0	48.4	36.0
Thailand	16 288	17.8	24 526	23.3	26 704	25.0	40.4	48.5	8.5	26.8	33.6	7.0
Turkey	2788	3.1	2536	2.8	5461	5.9	9.5	64.8	73.2	10.2	62.2	71.3
Turkmenistan (2015)	179	4.7	204	5.6	296	6.4	13.1	49.3	36.8	17.5	30.6	13.3
Uzbekistan	1037	5.1	1324	6.4	1545	6.3	24.3	39.3	15.4	22.6	21.1	1.6
**Oceania**	2468	4.6	1987	3.9	2503	4.8	21.6	1.4	23.0	16.5	4.3	20.7
Australia	2204	4.9	1726	4.0	2142	4.9	24.3	2.9	21.5	20.2	0.0	20.2
New Zealand (2016)	264	3.4	261	3.6	361	4.3	1.1	31.0	32.2	5.7	23.4	17.7
**Northern America**	31 193	5.1	26 467	4.1	34 805	5.2	16.4	10.9	27.2	21.7	1.9	23.7
Canada	3235	4.8	2667	3.7	3727	5.0	19.2	14.1	33.2	25.9	4.1	29.9
United States	27 958	5.2	23 800	4.2	31 078	5.3	16.1	10.6	26.5	21.3	1.9	23.2
**Southern America**	29 606	5.2	17 289	2.7	34 267	5.3	52.6	14.6	65.9	63.3	1.9	65.0
Argentina	1914	3.6	1172	2.0	2189	3.8	48.1	13.4	60.5	57.1	5.4	62.1
Bahamas (2015)	10	3.0	11	3.0	16	3.9	9.5	46.2	37.0	0.0	26.1	26.1
Brazil	10 899	5.2	5814	2.4	12 139	4.9	60.9	10.8	70.5	73.7	5.9	68.5
Chile (2018)	1348	5.7	712	2.9	1473	5.3	61.7	8.9	69.7	65.1	7.3	58.5
Colombia	1955	4.2	1394	2.5	2220	4.0	33.5	12.7	45.7	50.7	4.9	46.2
Costa Rica	393	7.5	269	5.0	438	6.9	37.5	10.8	47.8	40.0	8.3	31.9
Cuba	830	4.5	465	2.3	809	3.9	56.4	2.6	54.0	64.7	14.3	51.6
Dominican Republic (2018)	311	3.6	432	4.6	828	8.2	32.6	90.8	62.9	24.4	78.0	56.2
Ecuador	865	6.2	539	3.4	880	5.4	46.4	1.7	48.1	58.3	13.8	45.5
El Salvador (2018)	256	4.3	122	2.0	490	7.4	70.9	62.7	120.3	73.0	53.0	114.9
Guatemala	1526	16.2	521	4.5	1889	16.9	98.2	21.3	113.5	113.0	4.2	115.9
Jamaica (2014)	89	3.1	74	2.5	99	3.0	18.4	10.6	28.9	21.4	3.3	18.2
Mexico	6727	6.4	4176	3.5	7175	5.9	46.8	6.4	52.8	58.6	8.1	51.1
Nicaragua	320	7.7	172	3.9	577	12.0	60.2	57.3	108.1	65.5	43.7	101.9
Panama	170	4.5	126	2.9	215	4.8	29.7	23.4	52.2	43.2	6.5	49.4
Paraguay	135	2.7	118	2.1	176	3.0	13.4	26.4	39.5	25.0	10.5	35.3
Peru (2018)	1392	4.9	847	2.6	2093	6.0	48.7	40.2	84.8	61.3	20.2	79.1
Puerto Rico (2017)	311	5.0	196	2.7	402	6.1	45.4	25.5	68.9	59.7	19.8	77.3
Uruguay	155	2.9	129	2.2	159	2.8	18.3	2.5	20.8	27.5	3.5	24.0
**Northern Europe**	8946	4.0	7526	3.5	10 481	4.7	17.3	15.8	32.9	13.3	16.1	29.3
Denmark (2018)	482	3.8	372	3.2	633	5.1	25.8	27.1	51.9	17.1	29.2	45.8
Estonia	105	3.9	95	3.4	127	4.6	10.0	19.0	28.8	13.7	16.5	30.0
Finland (2018)	533	4.1	505	3.8	600	4.3	5.4	11.8	17.2	7.6	4.8	12.3
Iceland	23	4.0	16	2.7	22	3.5	35.9	4.4	31.6	38.8	13.3	25.8
Ireland (2018)	381	5.5	256	3.2	421	5.1	39.2	10.0	48.7	52.9	7.5	45.8
Latvia	175	4.7	107	2.6	125	3.2	48.2	33.3	15.5	57.5	38.0	20.7
Lithuania	230	4.0	170	2.9	245	4.5	30.0	6.3	36.1	31.9	11.8	43.2
Norway (2016)	279	3.0	232	2.5	386	3.8	18.4	32.2	49.8	18.2	23.5	41.3
Sweden (2018)	737	3.4	618	2.9	861	3.8	17.6	15.5	32.9	15.9	11.1	26.9
United Kingdom	6001	4.2	5155	3.7	7061	4.9	15.2	16.2	31.2	12.7	15.4	27.9
**Western Europe**	20 545	4.6	18 780	4.3	23 648	5.3	9.0	14.0	23.0	6.7	14.1	20.8
Austria	871	4.6	800	4.3	993	5.1	8.5	13.1	21.5	6.7	10.3	17.0
Belgium (2018)	938	3.9	861	3.6	1100	4.5	8.6	15.9	24.4	8.0	14.3	22.2
France (2016)	8639	6.4	7643	5.7	10 274	7.1	12.2	17.3	29.4	11.6	10.4	21.9
Germany	8168	4.0	7743	3.8	8872	4.4	5.3	8.3	13.6	5.1	9.5	14.6
Luxembourg	53	4.8	37	3.4	50	4.6	35.6	5.8	29.9	34.1	4.3	30.0
Netherlands	1139	3.0	939	2.6	1446	3.8	19.2	23.8	42.5	14.3	23.5	37.5
Switzerland (2018)	737	4.3	757	4.2	913	4.8	2.7	21.3	18.7	2.4	11.0	13.3
**Southern Europe**	22 974	5.7	17 530	4.4	25 033	6.3	26.9	8.6	35.3	25.7	10.0	35.5
Bosnia and Herzegovina (2016)	423	7.1	483	7.8	517	7.9	13.2	20.0	6.8	9.4	10.7	1.3
Bulgaria	715	4.8	646	4.5	584	4.0	10.1	20.2	10.1	6.5	18.2	11.8
Croatia	497	5.5	311	3.4	529	5.6	46.0	6.2	51.9	47.2	1.8	48.9
Cyprus (2018)	58	3.7	63	3.1	99	5.0	8.3	52.2	44.4	17.6	29.9	46.9
Greece	1480	5.3	783	3.0	1522	6.0	61.6	2.8	64.1	55.4	12.4	66.7
Italy (2017)	9263	5.9	6647	4.5	9798	6.4	32.9	5.6	38.3	26.9	8.1	34.9
Malta (2017)	31	3.4	20	2.1	31	3.3	43.1	0.0	43.1	47.3	3.0	44.4
Montenegro	48	4.5	60	5.8	49	4.4	22.2	2.1	20.2	25.2	2.2	27.5
North Macedonia	186	5.5	281	8.4	185	5.5	40.7	0.5	41.2	41.7	0.0	41.7
Portugal (2018)	1240	5.4	1043	4.4	1518	6.6	17.3	20.2	37.1	20.4	20.0	40.0
Romania	2840	7.5	1113	3.0	3380	9.0	87.4	17.4	100.9	85.7	18.2	100.0
Serbia	731	4.9	885	5.2	956	5.5	19.1	26.7	7.7	5.9	11.5	5.6
Slovenia	275	5.7	222	4.9	310	6.5	21.3	12.0	33.1	15.1	13.1	28.1
Spain	5187	5.3	4973	4.9	5555	5.5	4.2	6.9	11.1	7.8	3.7	11.5
**Eastern Europe**	17 921	3.7	12 423	2.7	19 435	4.1	36.3	8.1	44.1	31.2	10.3	41.2
Belarus (2018)	450	2.9	378	2.3	514	3.2	17.4	13.3	30.5	23.1	9.8	32.7
Czech Republic	908	4.0	633	2.8	873	3.8	35.7	3.9	31.9	35.3	5.1	30.3
Georgia	365	6.1	211	3.5	397	6.2	53.5	8.4	61.2	54.2	1.6	55.7
Hungary	859	4.2	512	2.5	937	4.7	50.6	8.7	58.7	50.7	11.2	61.1
Moldova (2018)	430	8.4	137	2.3	555	9.4	103.4	25.4	120.8	114.0	11.2	121.4
Poland	2060	2.8	1455	1.9	2455	3.3	34.4	17.5	51.2	38.3	16.4	53.8
Russia	10 430	4.2	6820	2.9	11 122	4.5	41.9	6.4	48.0	36.6	6.9	43.2
Slovakia	426	4.2	313	3.2	531	5.3	30.6	21.9	51.7	27.0	23.2	49.4
Ukraine	1993	2.8	1964	2.5	2051	2.8	1.5	2.9	4.3	11.3	0.0	11.3
**Africa**	8705	6.1	16 801	12.1	30 122	19.0	63.5	110.3	56.8	65.9	102.8	44.4
Egypt	6606	8.9	13 542	20.7	26 523	35.5	68.9	120.2	64.8	79.7	119.8	52.7
Mauritius	54	3.4	34	1.9	63	3.4	45.5	15.4	59.8	56.6	0.0	56.6
Morocco (2016)	211	0.7	586	2.1	1251	3.5	94.1	142.3	72.4	100.0	133.3	50.0
South Africa (2015)	1834	4.7	2639	6.3	2285	5.0	36.0	21.9	14.4	29.1	6.2	23.0

Abbreviations: WHO, World Health Organization; GBD, Global Burden Disease; GCO, Global Cancer Observatory; ASR, age‐standardized rate.

**FIGURE 2:**
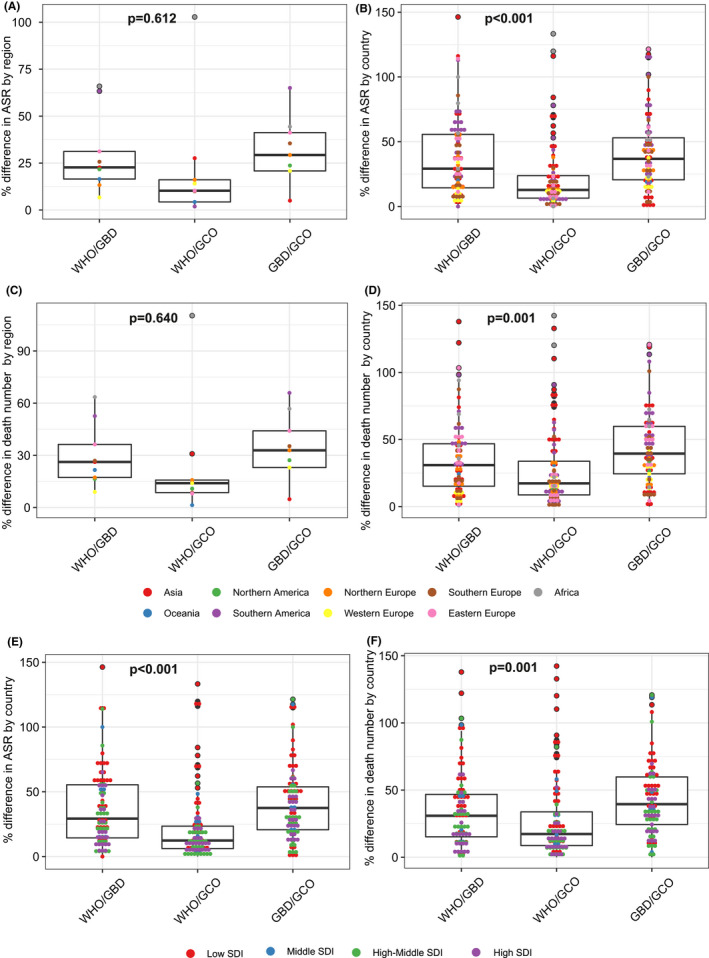
Comparisons of differences in age‐standardized rates and number of deaths of primary liver cancer mortality between the pairs among WHO, GBD and GCO by region, country and socio‐demographic index. WHO, World Health Organization; GBD, Global Burden Disease; GCO, Global Cancer Observatory; ASR, age‐standardized rate; SDI, socio‐demographic index.

Figure 2 shows the comparisons of the differences in ASR across the three data sources. In region level, the mean difference was found lowest between WHO and GCO (21.0%) than that between WHO and GBD (29.7%) and GBD and GCO (31.7%). The difference between WHO and GCO was mainly driven by estimates from Africa (102.8%), while the differences between WHO and GBD and between GBD and GCO were mostly driven by estimates from Africa (65.9% and 44.4%) and Southern America (63.3% and 65.0%) (Figure 2A). In country level, the mean difference in ASR was found significantly lower between WHO and GCO (21.7%) than that between WHO and GBD (37.8%) and between GBD and GCO (40.7%) (*p* < 0.001) (Figure 2B). The highest differences between WHO and GCO were found from 3 countries (all >100%): Libya, Egypt and Morocco (Table 2). As for the difference between the other two comparisons, 6 countries were found to have difference > 100%: Kyrgyzstan, Libya, Qatar, Guatemala, Moldova and Morocco between WHO and GBD and Kyrgyzstan, El Salvador, Guatemala, Nicaragua, Romania and Moldova between GBD and GCO (Table 2). While similar findings were also found for the differences in number of deaths in both region and country levels (Figure 2C,D), the absolute differences in number of deaths between WHO and GBD were found highest from countries in Asia, including Japan, South Korea, Thailand (Table 2). The differences between the three pairwise comparisons were also performed by SDI groups. Across the three comparisons for the differences in number of deaths and ASR, highest differences were found from Low SDI countries (Figure 2E,F).

In the sensitivity analysis, the estimated ASR was found similar to that in the main analysis for the same country from the same data source across the three data sources (Table A1). The difference in ASR between WHO and GCO was smaller than that between WHO and GBD and between GBD and GCO in the sensitivity analysis.

**FIGURE 3:**
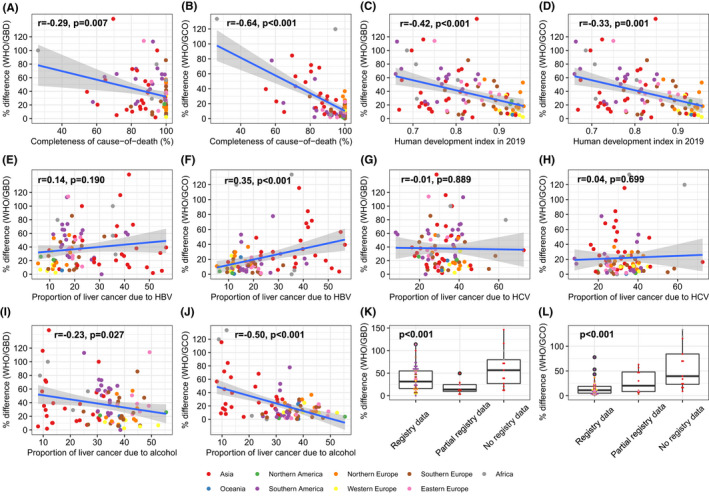
Correlations of differences in age‐standardized rates of primary liver cancer mortality from WHO, GBD and GCO with completeness of cause‐of‐death registration, human development index, proportion of liver cancer attributable to HBV, HCV and alcohol consumption, categories of data sources used by GCO. ASR, age‐standardized rate, WHO, World Health Organization; GBD, Global Burden Disease; GCO, Global Cancer Observatory; r represents the correlation coefficient.

In the analysis for the reasons of the differences across the three data sources (Figure 3), we found that completeness of cause‐of‐death registration showed a weak negative correlation with the difference in ASR between WHO and GBD (*r* = −0.29, *p* = 0.02) and strong correlation with the difference in ASR between WHO and GCO (*r* = −0.64, *p* = 0.02). Negative correlation was also found between human development index and the difference of ASR (*r* = −0.56 between WHO and GBD and *r* = −0.58 between WHO and GCO, *p* < 0.001). In contrast, positive correlation was found for the proportion of primary liver cancer attributable to HBV with the differences of ASR between WHO and GCO (*r* = 0.35, *p* < 0.001) but not between WHO and GBD (*r* = 0.14, *p* = 0.190). No correlation was found between the differences of estimates and proportion of liver cancer attributable to HCV. And the differences were negatively correlated with the proportion of liver cancer attributable to alcohol consumption. According to the data sources and methods used for the estimation by GCO, larger differences in ASR were found from countries without using registry data than those from countries based on complete or partial registry data (Figure 3).

**FIGURE 4:**
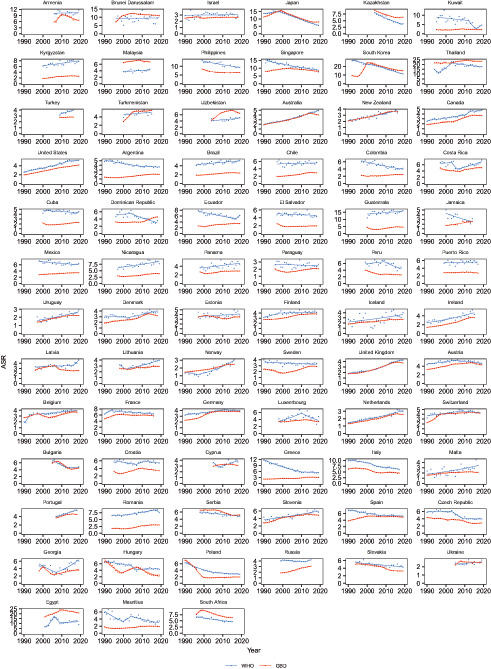
Joinpoint regression analysis of age‐standardized rates of primary liver cancer mortality for 75 countries from WHO and GBD, 1990–2019. WHO, World Health Organization; GBD, Global Burden Disease.

As for temporal trends of ASR from 1990 to 2019 between WHO and GBD for the 75 selected countries, similar trends from these two datasets were found from 35 countries in the most recent 10 years (Figure 4 and Table 3), including 14 countries with significant reduction, 18 countries with increase and 3 countries with stable trend. In contrast, opposite trends were found from 10 countries between these two data sources in the most recent 10 years (Table 3) with one from Asia: Uzbekistan (AAPC, 1.2 from WHO vs. −0.6 from GBD), four of them from Europe: Latvia (3.2 vs. −0.9), Sweden (−0.4 vs. 2.3), Cyprus (3.3 vs. −0.4), Poland (−1.2 vs. 0.8) and five of them from Southern America: Colombia (−2.2 vs. 1.4), Cuba (−0.5 vs. 2.1), Dominican Republic (−4.2 vs. 4.2), Jamaica (−3.7 vs. 3.2), Mexico (−0.7 vs. 0.7). The remaining 30 countries showed stable trends over the last 10 years in one dataset, but a reduction or increase in the other (Figure 4 and Table 3).

**TABLE 3:** Estimated Annual Average Percentage Change of primary liver cancer mortality in the last 10 years (2010–2019 or as indicated in the bracket) for 75 countries from WHO and GBD

	WHO		GBD	
	AAPC in the most recent 10 years (95% CI)	*p* value	AAPC in the most recent 10 years (95% CI)	*p* value
Asia				
Armenia	0.2 (−1.0 to 1.4)	0.749	−4.4 (−5.9 to −2.8)	<0.001
Brunei Darussalam	0.3 (−1.2 to 1.8)	0.686	−0.8 (−0.9 to −0.7)	<0.001
Israel (2018)	0.1 (−0.4 to 0.6)	0.703	0.3 (0.2 to 0.4)	<0.001
Japan	−5.5 (−5.9 to −5.1)	<0.001	−2.6 (−3.2 to −2)	<0.001
Kazakhstan	−5.2 (−5.8 to −4.7)	<0.001	−1.5 (−2 to −1.1)	<0.001
Kuwait	−4.8 (−18.2 to 10.9)	0.530	−1.4 (−1.9 to −0.9)	<0.001
Kyrgyzstan	1.3 (0.6 to 1.9)	0.001	0.2 (−0.2 to 0.7)	0.343
Malaysia (2014)	0.8 (−0.2 to 1.9)	0.101	−0.5 (−0.9 to 0)	0.031
Philippines	−1.7 (−2.2 to −1.2)	<0.001	−0.2 (−0.3 to 0)	0.051
Singapore	−1.9 (−2.2 to −1.6)	<0.001	−1.6 (−1.8 to −1.3)	<0.001
South Korea	−4.5 (−5.0 to −4.0)	<0.001	−2.8 (−3 to −2.6)	<0.001
Thailand	−1.3 (−1.9 to −0.7)	<0.001	−0.6 (−0.9 to −0.2)	0.003
Turkey (2016)	2.7 (1.4 to 4)	0.002	0.4 (0.1 to 0.7)	0.018
Turkmenistan (2015)	0.8 (−0.2 to 1.9)	0.117	0.4 (0 to 0.8)	0.032
Uzbekistan	1.2 (0.2 to 2.3)	0.026	−0.6 (−0.9 to −0.2)	0.001
Oceania				
Australia	3.2 (3.1 to 3.3)	<0.001	0.8 (0.2 to 1.3)	0.005
New Zealand (2016)	2.2 (1.7 to 2.6)	<0.001	1.5 (1.2 to 1.8)	<0.001
North America				
Canada	2.4 (−0.6 to 5.5)	0.118	1.5 (0.9 to 2)	<0.001
United States	1.4 (0.5 to 2.3)	0.002	1.6 (1.5 to 1.7)	<0.001
South America				
Argentina	−0.5 (−1.4 to 0.4)	0.301	1.1 (0.8 to 1.3)	<0.001
Brazil	0.8 (0.4 to 1.1)	<0.001	0.1 (−0.4 to 0.7)	0.601
Chile (2018)	0.2 (−0.4 to 0.8)	0.401	1.7 (1.2 to 2.1)	<0.001
Colombia	−2.1 (−2.6 to −1.6)	<0.001	1.2 (1 to 1.5)	<0.001
Costa Rica	3.2 (2.1 to 4.3)	<0.001	1.6 (0.9 to 2.3)	<0.001
Cuba	−0.5 (−1 to −0.1)	0.028	2.1 (1.8 to 2.3)	<0.001
Dominican Republic (2018)	−4.2 (−5.6 to −2.7)	<0.001	4.2 (3.3 to 5.2)	<0.001
Ecuador	0.4 (−2.9 to 3.9)	0.800	1.2 (0.9 to 1.4)	<0.001
El Salvador (2018)	−0.4 (−1.1 to 0.2)	0.181	0.7 (0.6 to 0.9)	<0.001
Guatemala	0.8 (0.1 to 1.4)	0.024	2.3 (1.5 to 3)	<0.001
Jamaica (2014)	−3.7 (−5.8 to −1.6)	0.003	3.2 (2.3 to 4.1)	<0.001
Mexico	−0.7 (−1.1 to −0.3)	0.001	0.7 (0.4 to 1)	<0.001
Nicaragua	1.5 (0.9 to 2.1)	<0.001	1.6 (0.6 to 2.6)	0.002
Panama	1.3 (0.3 to 2.3)	0.015	0.2 (0 to 0.5)	0.054
Paraguay	−0.4 (−1.1 to 0.4)	0.335	1.3 (0.7 to 1.8)	<0.001
Peru (2018)	−4.1 (−6.8 to −1.4)	0.006	−0.7 (−1.1 to −0.4)	<0.001
Puerto Rico (2017)	0.2 (−0.5 to 1.0)	0.498	−0.7 (−1.2 to −0.2)	0.007
Uruguay	2.3 (1.6 to 3.0)	<0.001	1.0 (0.8 to 1.3)	<0.001
Northern Europe				
Denmark (2018)	2.3 (1.3 to 3.4)	<0.001	0.9 (0 to 1.7)	0.043
Estonia	0.6 (−0.2 to 1.5)	0.140	1.3 (0.6 to 2)	<0.001
Finland (2018)	0.2 (−0.2 to 0.7)	0.255	0.4 (0.2 to 0.6)	<0.001
Iceland	1.7 (0.5 to 3)	0.009	0.6 (−0.1 to 1.3)	0.080
Ireland (2015)	2.6 (2.1 to 3.2)	<0.001	3.6 (3.2 to 4.1)	<0.001
Latvia	3.2 (0.3 to 6.1)	0.030	−0.9 (−1.6 to −0.1)	0.026
Lithuania	2.7 (2.1 to 3.4)	<0.001	0.7 (0.3 to 1.1)	0.002
Norway (2016)	5.1 (3.9 to 6.2)	<0.001	1.8 (1.6 to 2)	<0.001
Sweden (2018)	−0.4 (−0.7 to −0.1)	0.006	2.3 (1.2 to 3.5)	<0.001
United Kingdom	2.9 (2.3 to 3.5)	<0.001	1.6 (1.3 to 1.8)	<0.001
Western Europe				
Austria	−0.9 (−1.5 to −0.4)	0.002	−0.9 (−1.2 to −0.6)	<0.001
Belgium (2018)	0.8 (0.6 to 1.1)	<0.001	2.4 (1.9 to 2.9)	<0.001
France (2016)	−0.6 (−0.9 to −0.4)	<0.001	−0.3 (−0.4 to −0.1)	<0.001
Germany	−0.3 (−0.8 to 0.3)	0.324	0.1 (−0.1 to 0.2)	0.214
Luxembourg	−4.8 (−9.4 to 0)	0.050	−1.4 (−1.6 to −1.2)	<0.001
Netherlands	2.6 (2.3 to 2.9)	<0.001	1.2 (0.8 to 1.7)	<0.001
Switzerland (2018)	−0.6 (−1.8 to 0.5)	0.249	−0.2 (−0.7 to 0.3)	0.368
Southern Europe				
Bulgaria	−1.4 (−3.0 to 0.2)	0.078	−2.6 (−3.4 to −1.8)	<0.001
Croatia	−0.2 (−2.1 to 1.8)	0.868	−1.9 (−2.2 to −1.5)	<0.001
Cyprus (2018)	3.3 (0.8 to 5.9)	0.016	−0.4 (−0.6 to −0.1)	0.002
Greece	−0.8 (−1.8 to 0.3)	0.153	1.1 (0.1 to 2.2)	0.037
Italy (2017)	−1.5 (−2 to −1)	<0.001	−0.2 (−0.7 to 0.4)	0.533
Malta (2017)	2.4 (1.3 to 3.6)	<0.001	−0.3 (−0.7 to 0.1)	0.161
Portugal (2018)	3.1 (2.8 to 3.5)	<0.001	1.4 (1.2 to 1.6)	<0.001
Romania	0 (−1.3 to 1.4)	0.954	1.2 (0.5 to 1.9)	0.002
Serbia	−1 (−1.3 to −0.7)	<0.001	−1.6 (−1.9 to −1.3)	<0.001
Slovenia	1.7 (1.4 to 2.1)	<0.001	−0.1 (−0.6 to 0.5)	0.844
Spain	−0.4 (−0.9 to 0.1)	0.095	−0.3 (−0.4 to −0.2)	<0.001
Eastern Europe				
Czech Republic	−0.4 (−1.4 to 0.6)	0.407	−1.6 (−2.7 to −0.5)	0.005
Georgia	6.6 (2.9 to 10.4)	0.002	3.4 (2.4 to 4.5)	<0.001
Hungary	0.7 (−0.1 to 1.5)	0.093	−7 (−8.3 to −5.6)	<0.001
Poland	−1.2 (−1.6 to −0.7)	<0.001	0.8 (0.6 to 1)	<0.001
Russia (2015)	0.6 (−0.2 to 1.5)	0.156	3.2 (2.7 to 3.6)	<0.001
Slovakia	−0.9 (−1.3 to −0.6)	<0.001	−2.5 (−3.3 to −1.6)	<0.001
Ukraine	0.8 (−0.2 to 1.8)	0.122	−0.3 (−0.8 to 0.3)	0.263
Africa				
Egypt	0.8 (−2.2 to 3.9)	0.580	−1.6 (−1.9 to −1.2)	<0.001
Mauritius	−3.2 (−4.7 to −1.8)	<0.001	0.2 (−0.6 to 0.9)	0.637
South Africa (2015)	−2 (−2.3 to −1.6)	<0.001	−2.1 (−2.5 to −1.7)	<0.001

Abbreviations: WHO, World Health Organization; GBD, Global Burden Disease; AAPC, Average Annual Percentage Change is a summary measure of the trend over a pre‐specified fixed interval. *p* less than 0.05 was considered as statistically significant.

**FIGURE 5:**
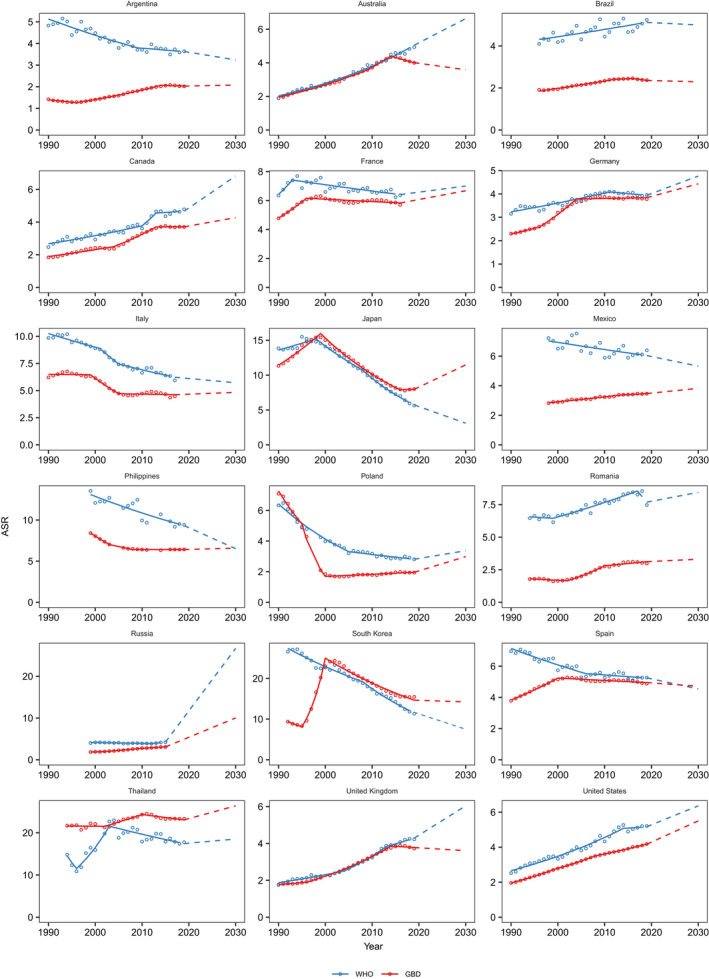
The predicted age‐standardized rates of primary liver cancer mortality up to 2030 in 18 selected countries from WHO and GBD. WHO, World Health Organization; GBD, Global Burden Disease.

Of the 18 countries with the highest average number of primary liver cancer deaths projected up to the year 2030, only South Korea showed a consistent reduction in liver cancer mortality from both WHO and GBD (Figure 5). Our projections from both data sources suggested that the ASR of liver cancer death would continue to increase or stabilize in another 10 countries. Interestingly, our projection from WHO indicated the ASR would decrease in Argentina, Italy, Japan, Mexico and Philippines, while projections from GBD suggested continuous increase up to 2030. In contrast, the ASR in Australia and United Kingdom would increase based on estimates from WHO, whereas it would decrease based on estimates from GBD.

## 4 DISCUSSION

This study compared the ASR of primary liver cancer deaths in 92 countries from WHO, GBD and GCO in the most recent year. The estimates in ASR were quite consistent across the three data sources while a lower difference of ASR was found between WHO and GCO than those between the other two comparisons. The differences of ASRs between WHO and GBD and between WHO and GCO were negatively associated with completeness of cause‐of‐death registration, HDI and proportion of liver cancer because of alcohol consumption, but the positive association of difference in ASR with the proportion of liver cancer because of HBV was only found between WHO and GCO, and no association of difference in ASR was found with the proportion of liver cancer because of HCV. As for the temporal trend in the most recent 10 years between WHO and GBD, similar trends were found from 35 countries between these two data sources and opposite trends were found from 10 countries between them.

Although the three data sources provided estimates in number of deaths and GBD and GCO also calculated ASR based on their standard populations, we calculated the ASR using direct standardization based on the same world standard population. This allows us to compare the differences of primary liver cancer mortality from different countries and regions across the three data sources in a more sensible and coherent way as suggested by previous studies.^3^

Of the three major data sources for the estimates of primary liver cancer mortality, the most similar estimates of ASR were found from WHO and GCO. Together with their available original input data and methodology applied,^4^, ^14^ our study therefore suggested these two data sources provided more reliable estimates of primary liver cancer deaths. Our study also highlighted the differences in ASR between WHO and GCO were strongly correlated with the completeness of cause‐of‐death registration data, where the largest variations could be found from countries with lower completeness of cause‐of‐death registration data. This was further supported by the source of original data used for the GCO estimates, showing the variations were found higher from countries using partial registry data or countries without using registry data. These results suggested the differences in ASR between WHO and GCO were mostly explained by countries with poor vital registry system. Some of the reasons for incomplete vital registration are a poor legal framework to support registration, the lack of a sound process of notification of vital events to the registry system, and a lack of coordination among multiple institutions facilitate data transfer and compilation.^20^ Therefore, our study highlighted the importance to set up strong and complete vital registration system for countries without good registration system, which would allow the clinicians and researchers to have a better understanding of the burden of mortality and prioritize health policy in these countries.

Compared to the availability of input data used for each country and methodology applied by WHO and GCO, the GBD study did not provide original data for their input sources and used complex methodology for estimation.^13^ These two major reasons make us harder to explain where the variations come from. Additionally, the GBD study is producing its own population, which differ from those of the WHO Population Division.^11^ This means that GBD estimates of numbers of deaths will differ systematically from those produced by WHO and GCO even when based on similar death rates. However, we found the estimates of ASR from GBD were generally comparable with those from WHO and GCO, suggesting the estimates from GBD might be also based on the WHO mortality data for most of the countries included in this study. The major differences between GBD and WHO were found from countries in Africa and Southern America. Reasons for some of these are known, which could arise from differences in input data and its adjustments. As input data, the GBD has used vital registration data as well as cancer registry, verbal autopsies and peer‐reviewed literature in the methods while WHO has only used vital registration data. As for adjustment, the GBD has adjusted a large set of aggregated causes and uninformative causes of liver cancer, whereas no adjustment was made by both WHO and GCO.^21^ In addition, the highest absolute difference in number of deaths between GBD and WHO was found from countries in Asia. Although reasons for the differences from these countries remained unknown, it reflected the variations of age‐specific estimation between GBD and WHO.

While the differences in ASR between GBD and WHO and between GBD and GCO were higher than that between WHO and GCO, it is the only data source to provide estimates by aetiology and year consistently.^13^ This would allow policy makers and clinicians to have a better understanding of the attributable risk of liver cancer mortality in each country and their temporal trends. The fact that these independent institutions make important effort to come up with global burden of primary liver cancer must be welcomed and appreciated. It is beneficial that not a single institution claims full authority on mortality estimates, which allows further discussion methods and provides improvement of estimates for relevant public health indicators.

Despite the variation of ASR in the most recent years, 35 out of 75 countries showed a similar trend of mortality rate in primary liver cancer in the most recent 10 years between WHO and GBD. Of the 35 countries, 18 of them showed increasing trends of primary liver cancer mortality over the period, highlighting the continuing increased burden of primary liver cancer from countries in Oceania, North America and Europe as reported previously.^9^, ^10^ Multiple risk factors other than HBV and HCV are possibly the cause of the increasing trend from these countries, including nonalcoholic fatty liver diseases (NAFLD) associated with the global epidemic of obesity and type 2 diabetes becoming the most rapidly growing contributor.^22^, ^23^ Another 14 countries showed a consistent reduction of primary liver cancer mortality in the most recent 10 years from both WHO and GBD study, including Japan, Kazakhstan, Peru, Slovakia, South Africa. Most to these countries have high burden of HBV infections in the past, but because of the success of universal HBV vaccination programme as well as the introduction of HCV screening and administration of effective antiviral treatment for HBV and HCV.^24^, ^25^, ^26^

Of significance, opposite trends in five countries from South America, four countries in Europe and one country in Asia between the WHO and GBD data raised concerns of the actual patterns of liver cancer mortality from these countries. For seven of these ten countries, the estimates from WHO suggested a reduction of primary liver cancer mortality in the last 10 years while substantial increase was found from GBD in the same period. Such patterns are consistent with previous study. For instance, the reduction in ASR from WHO in this study was in line with previous study‐based WHO data for Sweden and Poland,^27^ suggesting the ongoing reduction of liver cancer mortality from these countries. Similarly, the studies based on GBD data had suggested increasing trend over the periods for these two countries.^10^, ^13^ The increasing trends of mortality from GBD data could be partially explained by the approach of modelling the incidence and mortality ratio, where increasing incidence was observed from recent years because of the high prevalence of NAFLD in these countries.^28^ In our study, Latvia and Cyprus were the only countries showing increasing trend from WHO and reduction from GBD. Such findings are consistent with other studies based on previous data from WHO and GBD.^29^, ^30^ However, the reason for such opposite direction of trends remains unclear for these countries. It is therefore important to know the contribution of each data sources used for the final estimates in GBD modelling data. However, such data is not publicly available for researchers.

Our projections of a decrease and increase of primary liver cancer mortality up to 2030 for Japan and the United States, respectively, were consistent with previously reported projections until 2020 based on the WHO mortality data and the same method.^9^ Our trends were also generally consistent with those obtained in a different study using the age‐period cohort model on GBD data for liver cancer incidence.^31^ Our projections suggested that mortality of primary liver cancer will continue or start to increase for another 10 countries based on both the WHO and GBD data, though these estimates were based on simplistic assumptions about the continuation of current trends and the model did not account for the recent introduction of HCV screening and the administration of effective treatment, which was already shown to impact cirrhosis and primary liver cancer incidence in studies from France,^32^ Australia,^33^ and the United States.^34^ More importantly, our results highlight how using the varying trends from WHO and GBD to predict the future burden of primary liver cancer mortality could result in opposite directions of change for these countries in the future, as seen in our projections for Argentina, Italy, Japan, Mexico, Philippines, Australia and United Kingdom. Therefore, caution should be taken for the choice of the source data for trend analysis and burden estimation.

The main strength of this study is the use of the most comprehensive mortality data from WHO, GBD and GCO to assess and compare the current burden of primary liver cancer mortality. Although the GBD and GCO provided mortality data for 195 and 185 countries or territories worldwide, only countries with available mortality registration data from the WHO were selected in this study. This brings to one of the limitations of this study is that countries without good quality registration data from vital registry were not included in this study. Therefore, this study did not analyse the burden and trend of primary liver cancer in some countries with high burden of liver cancer. This includes China and India, which in total accounts for about two thirds of primary liver cancer mortality worldwide. Another limitation is that while the correlation with potential factors leading to differences between the datasets were explored, specific reasons for the differences of ASR from GBD with that from the other two data sources could not be analysed because of lack of availability of detailed input data sources and modelling methods. However, the GCO has provided the detailed information of the data sources for each country, which allows us to identify the major differences of ASR between WHO and GCO were from countries with partial or no registry data. The other limitation of this study was that the estimates from GCO was only available for year 2020, while the estimates from WHO was from year 2019 or earlier. However, in the sensitivity by including countries from WHO with estimates in year 2020, the estimated ASR was found similar to that in the main analysis for the same country from the same data source. This suggests the inclusion of estimates from different year in this study did not result in the differences of ASR across the data sources. Finally, although our projections of primary liver cancer mortality were generally consistent with previous studies, our model was only based on simple assumptions about the continuation of current trends, and it did not consider the impact of the recent introduction of HCV screening or the administration of effective treatment. Therefore, more complex models are required for more accurate predictions. However, this would be enough for this study to show that the different choice of data sources for projections could result in opposite directions of change for some countries in the future.

In conclusion, while the estimates of primary liver cancer mortality were quite consistent across the three data sources for 92 countries overall, most similar estimates were found between WHO and GCO. Opposite trends of ASR between WHO and GBD were identified from one Asian country (Uzbekistan), four European countries (Latvia, Sweden, Cyprus and Poland) and five Southern American countries (Colombia, Cuba, Dominican Republic, Jamaica and Mexico). Caution should be taken for the choice of the source data for trend analysis and projection.

## CONFLICT OF INTEREST

The authors declare that they have no known competing financial interests or personal relationships that could have appeared to influence the work reported in this paper.

## FUNDING STATEMENT

This research did not receive any specific grant from funding agencies in the public, commercial or not‐for‐profit sectors.

## APPENDIX A

**Table A1:** The comparison of primary liver cancer death in number of deaths and ASR across the three data sources. Only countries with deaths reported in year 2020 from WHO dataset were included

Region/country	WHO		GBD		GCO		% difference in ASR		
	No. of death	ASR	No. of death	ASR	No. of death	ASR	WHO/GBD	WHO/GCO	GBD/GCO
Asia									
Kazakhstan	659	3.5	1116	6.3	988	5.1	58.7	38.4	21.6
Qatar	31	3.0	91	14.2	54	5.4	131.0	58.4	89.8
Singapore	563	7.7	658	8.0	1270	13.6	4.3	55.5	51.4
Oceania									
Australia	2192	4.7	1726	4.0	2142	4.9	16.6	3.7	20.3
Southern America									
Costa Rica	371	5.8	269	5.0	438	6.9	13.9	18.1	31.9
Ecuador	744	4.4	539	3.4	880	5.4	25.9	19.7	45.0
Guatemala	1632	14.5	521	4.5	1889	16.9	105.1	15.1	115.6
Mexico	6771	5.5	4176	3.5	7175	5.9	45.3	7.1	52.0
Northern Europe									
Estonia	132	4.7	95	3.4	127	4.6	30.5	1.6	28.9
Iceland	16	2.5	16	2.7	22	3.5	8.1	34.5	26.6
Latvia	184	4.6	107	2.6	125	3.2	55.4	35.4	21.1
Lithuania	235	4.1	170	2.9	245	4.5	32.7	10.2	42.5
Western Europe									
Austria	847	4.3	800	4.3	993	5.1	1.0	16.6	17.6
Germany	8457	4.1	7743	3.8	8872	4.4	6.8	8.2	15.0
Netherlands	1268	3.2	939	2.6	1446	3.8	23.5	15.8	38.9
Southern Europe									
Serbia	646	4.3	885	5.2	956	5.5	18.9	24.3	5.5
Slovenia	301	6.2	222	4.9	310	6.5	24.1	4.0	28.0
Spain	5021	4.7	4973	4.9	5555	5.5	3.7	15.6	11.9
Eastern Europe									
Czech Republic	872	3.7	633	2.8	873	3.8	27.4	1.6	28.9
Georgia	457	7.6	211	3.5	397	6.2	73.4	20.9	54.6
Africa									
Mauritius	55	3.0	34	1.9	63	3.4	45.8	12.2	57.2
